# Application of CO_2_ clearance to estimate cardiac output in patients with venoarterial extracorporeal membrane oxygenation

**DOI:** 10.1186/s13054-023-04723-z

**Published:** 2023-11-10

**Authors:** Zhipeng Xu, Tong Li, Jueyue Yan

**Affiliations:** Department of Critical Care Medicine, The First Affiliated Hospital, Zhejiang University School of Medicine, No. 79 Qingchun Road, Hangzhou, 310003 Zhejiang Province China

## Dear Editor,

Patients supported by VA-ECMO are characterized by end-organ hypoperfusion due to low CO (cardiac output) and hypotension. The measurement of CO in these patients plays an important role in the evaluation and treatment of the disease. However, several hemodynamic monitoring methods used to assess the patient's own CO have not been validated, and their effectiveness is still controversial. In addition to using ultrasound to estimate CO, other methods, such as Pulmonary artery catheter (PAC), Transpulmonary thermodilution (TPTD), and Arterial pressure waveform analysis (APWA) have some defects in VA-ECMO patients, which makes it impossible to estimate CO normally [1].

**Fig. 1:**
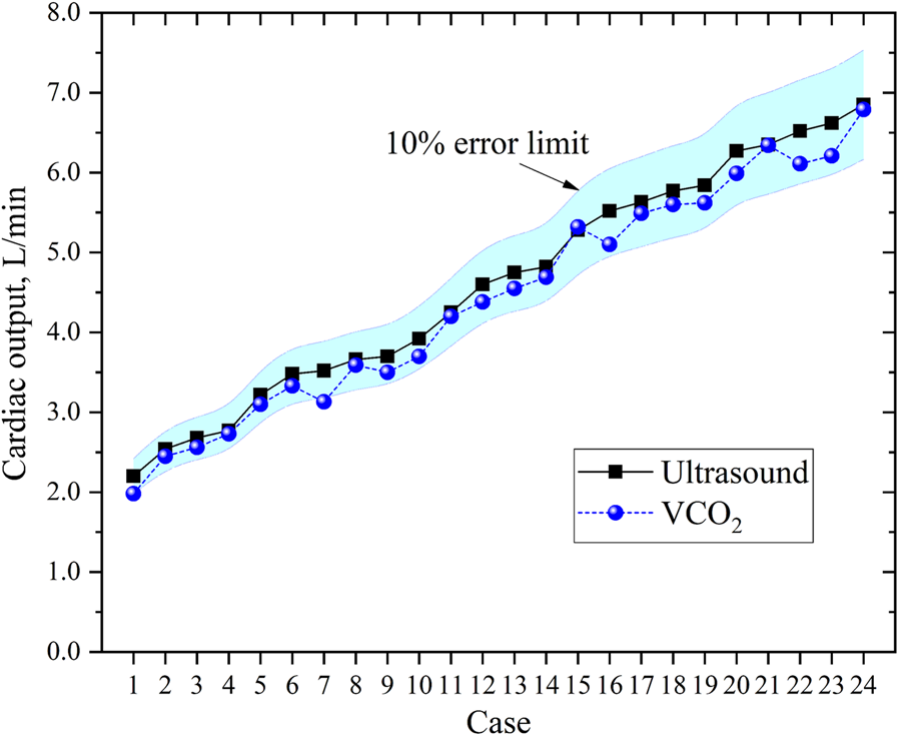
Comparison of cardiac output measured by VCO2 and Ultrasound

The Fick equation applied to CO_2_ indicates that the CO_2_ clearance (equivalent to CO_2_production in a steady state) equals the product of cardiac output by the difference between CCO_2_ in mixed venous blood (CvCO_2_) and arterial blood (CaCO_2_): VCO_2_ = CO × (CvCO_2_−CaCO_2_); CO = VCO_2_/(CvCO_2_−CaCO_2_) [2]. Our group has observed that this applies in patients with VA-ECMO who have partial cardiac output and no intracardial shunts or valvular regurgitation (with blood supplied from the right radial artery). A total of 24 eligible patients with VA ECMO were observed by our team. VCO_2_ was measured with the use of a Mindray SV850 ventilator. The ventilator has a built-in CO_2_ monitor, which uses the mainstream absorptiometry method to accurately monitor the concentration of CO_2_ in the exhaled gas, and the VCO_2_ is measured according to the minute ventilation. Regarding mixed venous blood, we obtained it through a pulmonary artery catheter. All blood gas samples were analyzed by an ABL90 FLEX blood gas analyzer. The Mchardy–Visser formula was used to calculate the CCO_2_ results. Because the formula was corrected for HB, PH, and SO_2_, the results were highly reliable [3]. CO was estimated by having a professional sonographer use ultrasound equipment to measure CO, and the patient was also subjected to arterial-venous blood gas analysis and VCO_2_ recording. We compared the CO measured by the two methods and found that the error between them was less than 10% (Fig. 1).

The method of measuring CO by VCO_2_ has its unique advantages over the method of measuring CO by ultrasound. Its data are collected by precision instruments, which eliminate the interference of human factors.CO can be assessed relatively accurately by a simple ventilator setting and arterial and venous blood gas analysis. It makes it easier and faster for ICU doctors to evaluate the cardiac function of patients. However, we must admit that there are some limitations in estimating CO with VCO_2_. The human heart is subject to neural, and humoral regulation. Cardiac output is adjusted to meet systemic metabolic demands in response to changes in basal metabolic rate. As the metabolic rate changes, the CO_2_ produced by the body will also change, thus changing the VCO_2_ and CCO_2_. Using VCO_2_ to assess CO requires that the body's metabolic rate is stable to improve the reliability of the results. In the included observation cases, due to the operation of VA ECMO, we did adequate sedation and analgesia during the measurement of CO to avoid the huge fluctuations in the body's metabolic rate in a short period and reduce errors.Also we need to acknowledge that the sample size of the present study is relatively small, and the data presented in this paper are primary observations. Larger and deeper clinical trials are already underway. Our team believes that routine VCO_2_ measurement in patients with VA-ECMO is beneficial to disease monitoring and accurate treatment.

## Data Availability

The datasets used and/or analyzed during the study are available from the corresponding author upon reasonable request.
